# Proposal of a Modular Classification System for Direct Dental Resin Composites Based on Clinical Applications

**DOI:** 10.3390/polym17050564

**Published:** 2025-02-20

**Authors:** Philippe Francois, Mathieu Izart, Timothy Fasham, Yasmine Smail, Marie Jannot, Stéphane Le Goff, Fleur Beres, Max Troizier-Cheyne, Sara Bergman, Christian Moussally, Sarah Abdel-Gawad, Elisabeth Dursun, Romain Ceinos, Elisa Caussin, Jean-Pierre Attal

**Affiliations:** 1 Faculty of Dental Surgery, UMR-1333 Oral Health, Paris-Cité University, Bretonneau Hospital, 1 rue Maurice Arnoux, 92120 Montrouge, France; 2 Faculty of Dental Surgery, UMR-1333 Oral Health, Paris-Cité University, Charles Foix Hospital, 1 rue Maurice Arnoux, 92120 Montrouge, France; 3 Faculty of Dental Surgery, UMR-1333 Oral Health, Paris-Cité University, 1 rue Maurice Arnoux, 92120 Montrouge, France; 4 Faculty of Dental Surgery, UMR-1333 Oral Health, Paris-Cité University, Henri Mondor Hospital, 1 rue Maurice Arnoux, 92120 Montrouge, France; 5 Faculty of Dental Surgery, UMR-1333 Oral Health, Côte d’Azur University, 5 rue 22ème BCA, 06300 Nice, France

**Keywords:** resin composite, flowable, viscous, layering, bisphenol-free, bulk-fill, fiber-reinforced, highly filled, simplified color integration, ion-releasing

## Abstract

The adhesive–resin composite pair has been the cornerstone of direct restorations in dentistry for many years. Resin composites are traditionally classified in three ways based on their inorganic structure, their organic composition and their viscosity. While these classifications have long been associated with the optical, mechanical, and clinical properties of resin composites, recent studies indicate that this classification is not always valid. In recent years, a significant expansion of the range of clinical resin composite families has occurred, each with varying degrees of validation through in vitro and clinical studies. As a result, new resin composites with distinct structures, viscosities, and clinical indications have emerged. Despite this progress, a formal classification of the clinical features of all resin composites is still lacking, leading to terminological inconsistencies in research and potential confusion among clinicians. This brief review, supported by an exhaustive search of the dental literature, proposes a new clinical classification system for resin composites based on their key clinical features to help clinicians and researchers easily identify the key clinical characteristics of formulations. This modular classification, encompassing eight main families and 14 characteristics, is particularly suited to future developments, as current trends aim to simplify procedures by integrating multiple formulations into single products.

## 1. Introduction

In recent years, both the scientific literature and the dental industry have highlighted a notable trend: the simplification of clinical procedures and the development of more versatile products [1,2]. Dental adhesives and resin composites are widely regarded as the gold standard for direct restorations [3,4,5,6]. Despite their ability to achieve reliable long-term clinical performance, significant potential for improvement remains [6,7], although the longevity of a restoration is much more influenced by factors specific to the patient and the practitioner than by factors related to the materials used [7]. Current advancements are focused on enhancing user-friendliness and integrating smart behaviors into these materials [8,9].

Classifications are often of great help to clinicians and researchers aiming to choose or understand how a resin composite works or should be used. Existing classifications based on the inorganic composition, organic structure, or viscosity have provided valuable guidance in the past but are now of limited clinical utility [9,10,11,12,13,14]. In fact, classifications based on inorganic filler microstructures (size, weight or distribution) [15,16,17], viscosity [11,18,19,20,21,22,23] or even monomer composition [24,25,26] do not or can no longer predict the main clinical and physical characteristics of a resin composite. The aim is not to question the relevance of these classifications for the development or elaboration of new formulations, but to propose one that is closer to the needs of a clinician. The need for a classification closer to the main clinical characteristics or interest of these resin composites has already been highlighted by authors [27,28]. Surprisingly, the proposed classification of resin composites based on their clinical practicalities has never yet been implemented in a scientific article, despite being used in numerous titles, abstracts and contents of international publications.

Recent advancements have led to the emergence (or reintroduction) of various resin composites, each offering unique characteristics, including but not limited to bulk-fill capabilities [29], glass-fiber reinforcement [30], flowable resin composites with an increased filler content [22], simplified color integration [31], or even ion-releasing properties [32]. Furthermore, a single product can integrate multiple characteristics within the same formulation, making its identification and selection more complex for researchers and clinicians. The growing overlap of these features can create challenges for practitioners in clinical decision-making. However, a thorough understanding of the key parameters governing these characteristics is crucial for their effective application. To facilitate this, clinical diagrams have been also developed in this article to provide clear explanations.

Therefore, the objective of this brief review, along with the proposed classification system, which is based on an exhaustive literature review and the authors’ expertise, is to present a classification system rooted in the key clinical characteristics of contemporary resin composites. By outlining these primary features, a more structured approach to classifying resin composites can be established, even when a resin exhibits multiple characteristics.

## 2. Current Limitations of Direct Dental Resin Composite Classifications

Dental resin composites can be classified based on their unique formulations, which are specifically designed to meet the diverse needs of various applications, including the fabrication of direct restorations of various extents combined with the use of a dental adhesive. These materials share a common structure, consisting of a polymeric matrix (usually a blend of dimethacrylate resins) reinforced with glass fillers. Additionally, a silane coupling agent is included to ensure proper adhesion between the fillers and the matrix. Other elements, such as pigments, preservatives and polymerization agents, are also present to facilitate or regulate the polymerization process [9,33]. After the polymerization reaction, the degree of conversion of a resin composite is between 40% and 70%, leading to the persistence of unreacted monomers (and other chemical molecules) in the final material [34,35,36].

**Figure 1:**
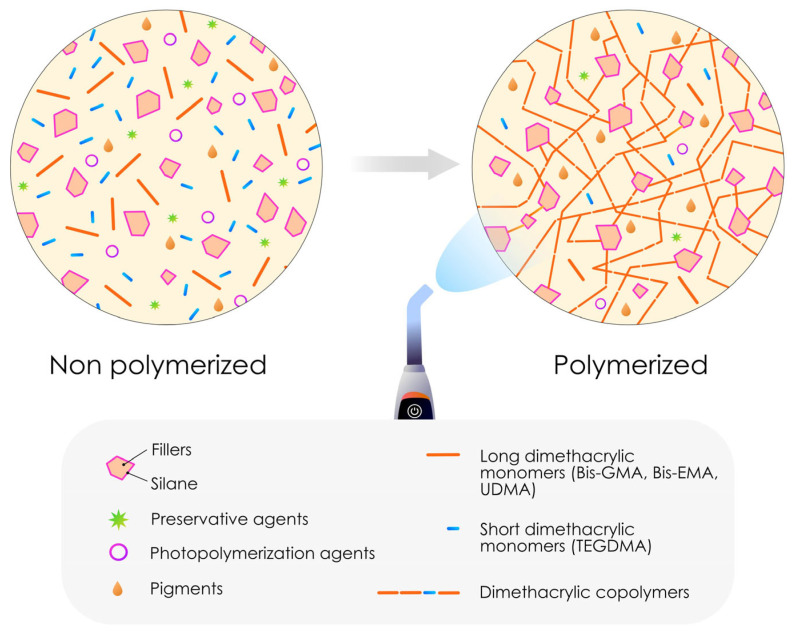
Schematic representation of the structure of a dental resin composite. The majority of these resin composites are therefore composed of inorganic fillers embedded in an organic polymer matrix, with a coupling agent called silane acting as the binder between the two. In the context of direct resin composites, the degree of conversion is never 100%, and thus residual free monomers remain present after polymerization.

Due to their composition and chemical, physical and aesthetic imperatives, the most common forms of most dental resins must be laminated in 2 mm layers to ensure proper polymerization. The 2 mm polymerization depth is a general characteristic of dental composite resins, except when specific properties, such as bulk-fill capabilities or self-curing mechanisms, are present, which can modify this parameter [37,38,39,40]. Figure 1 shows a schematic representation of the structure of a classic resin composite before and after polymerization.

### 2.1. Classification Based on the Filler Size and Distribution

Historically, resin composites have evolved through changes in filler size and distribution [9,10]. Thus, a classification based on the filler structure (particularly their size) of resin composites is currently the most widely used method in the dental literature [9,10] and represents a chronology of the development of these resins over the last 50 years. The first type of resin composite, known as “macrofill”, has large particles (10–50 µm) that are difficult to polish [41,42]. “Microfill” resin composites have been developed to improve aesthetics, although their particle size is closer to the nanoscale. Prepolymerizations containing these small fillers have been incorporated to increase the filler ratio, which is limited by the drastic increase in specific surface area induced by the use of these small fillers, but this approach is insufficient to obtain the correct mechanical properties [42,43].

**Figure 2:**
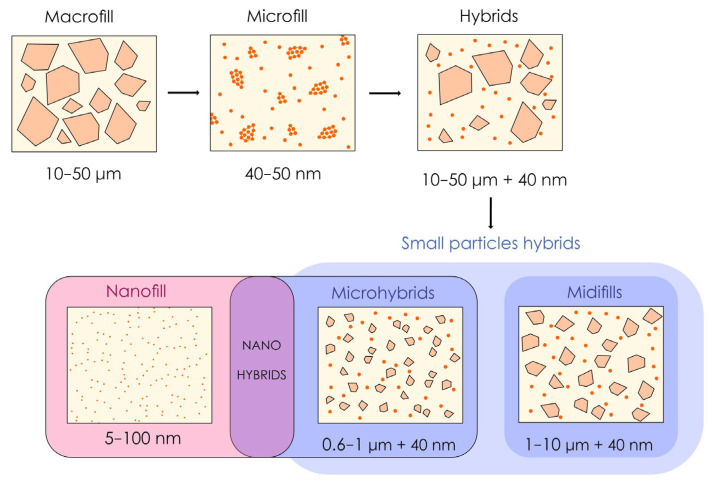
Illustration inspired and slightly modified by the authors of “Resin composite–state of the art”, 2011 [9]. The progressive evolution of resin composite formulations has involved a gradual reduction in the average size of fillers, the inclusion of prepolymerized fillers (which gradually tend to disappear) and the incorporation of nanofillers. The three most common families used today are microhybrids, nanohybrids and nanofills.

This approach is how modern resin composites, some of which are still used clinically, were developed, with the creation of hybrids (midifill and microhybrid resin composites) designed to combine the wear-resistance properties of macrofill resin composites with the polishability of microfill resin composites [1,9]. The latest development, “nanofill” resin composites with particle diameters as small as 5 nanometers, uses exclusively nanoscale particles and leads to the creation of “nanohybrids” if combined with larger fillers [1,9]. Figure 2 shows the gradual evolution that has led to the development of the current dental resin composites in terms of the structure and filler distribution.

Microhybrid, nanohybrid and nanofill resin composites are among the most widely represented families on the market today. However, in terms of the clinical performance and mechanical or optical properties of these formulations, only a weak correlation exists between their structure and their characteristics [5,16,23,42,44,45,46]. This finding raises the question of the clinical relevance of this classification, although it is still of interest for research purposes.

### 2.2. Classification Based on the Monomer Composition

Although less commonly used, resin composites can also be classified according to their monomeric composition [13,47]. Indeed, as far as the monomer composition is concerned, several decades of continuous research were needed for credible evolutions to bisphenol-A-glycidyl methacrylate (bis-GMA), triethylene glycol dimethacrylate (TEGDMA) or urethane dimethacrylate (UDMA). These monomers are still combined in resin composites for a synergetic effect. Bis-GMA provides high mechanical strength but is very viscous, whereas TEGDMA acts as a low-viscosity diluent, improving handling and polymerization [48,49,50]. UDMA adds flexibility and enhances wear resistance. Together, they balance rigidity, flexibility, and polymerization-induced shrinkage, creating a durable and stable network. This synergy ensures the ease of manipulation, mechanical durability, and aesthetic outcomes [48,49,50].

**Figure 3:**
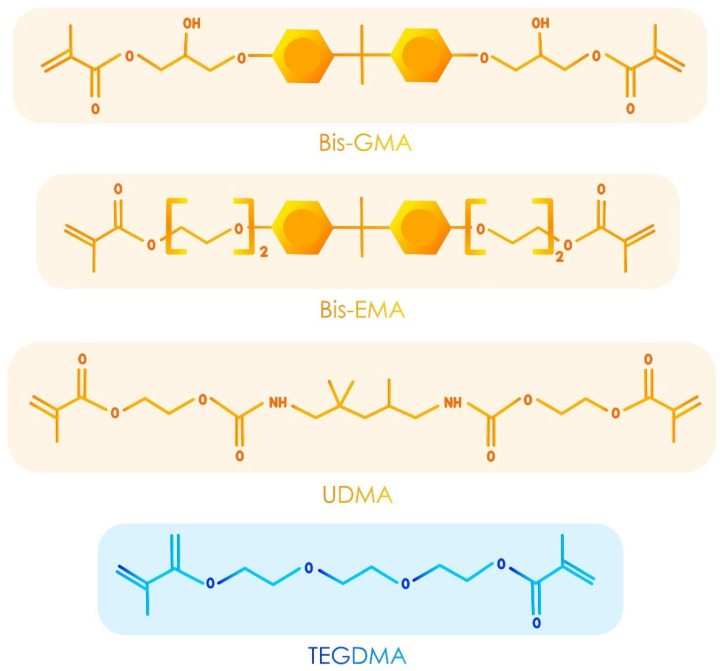
Developed formulas of the main monomers found in the resin composites.

These monomers have undergone evolutionary rather than revolutionary development, with the aim of improving some of their properties or reducing the degree of polymerization stress generated by their use [14,51,52]. The most striking monomer developments derived from these monomers include Bis-EMA development (an ethoxylated version of Bis-GMA with lower viscosity, reduced hydrophilic behavior, reduced shrinkage stress and enhanced translucency) [50,53,54] and stress relievers that modified UDMAs initially found in bulk-fill resin composites, which will be described later [55,56]. Figure 3 shows the developed formulas of the main monomers found in the resin composites.

More disruptive attempts have been made with the evolution of conventional acrylate matrices, such as ring-opening matrices (siloranes) [57,58], inorganic matrices (ormocers) [59] and monomers incorporating lyophylated acids (compomers) [32]. However, as with the previous classification, classifying resin composites based on their monomeric composition alone appears too simplistic and too unpredictable in terms of clinical, mechanical or optical performance [15,25,60,61,62].

### 2.3. Classification Based on Viscosity

**Figure 4:**
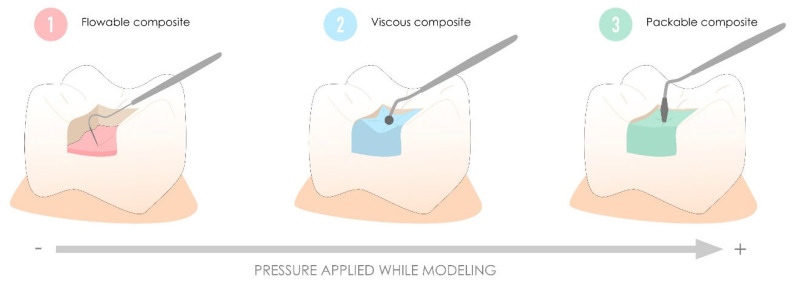
Major indications for resin composites as proposed by the historical classification based on viscosity. Flowable resin composites are primarily used for their self-leveling and self-spreading properties in thin layers and as liners. In contrast, viscous and packable resin composites are better suited for larger cavities subject to occlusal stress. Packable resin composites, in particular, are designed to work with firm pressure, similar to the handling of amalgams.

This classification divides resin composites into three families: flowable resin composites with self-levelling and self-spreading properties, viscous resin composites with creamy manipulation, and packable resin composites with handling properties similar to those of amalgams [63,64]. In contrast to packable and viscous resin composites, which have similar properties and clinical outcomes, despite different handling methods [65,66,67], flowable resin composites obtained by reducing the filler ratio have inferior mechanical and optical properties, resulting in a lower longevity than resin composites with other viscosities in areas subject to high occlusal stress, explaining their main use as liners [68,69,70]. Figure 4 illustrates the major indications for resin composites proposed based on this historical classification.

Although these generalities have held true for many years, we can now observe that these properties are no longer observed and that in certain in vitro [71] or clinical studies [11,20,72], flowable resin composites show comparable or inferior results in terms of wear to viscous resin composites [73]. Moreover, packable resin composites have all but disappeared from the market, as clinicians have failed to make them popular in terms of handling.

Finally, subfamilies of this classification are sometimes proposed for flowable and viscous resin composites:

- high-flow, low-flow and superlow flowable resin composites according to their spreading capacity;
- high-viscosity viscous resin composites (different from packable resin composites), and low-viscosity viscous resin composites with creamier consistency.

However, the use of this specific terminology remains highly inconsistent and subjective and cannot be applied efficiently to characterize product viscosity.

### 2.4. Classification Based on the Location of Use

**Figure 5:**
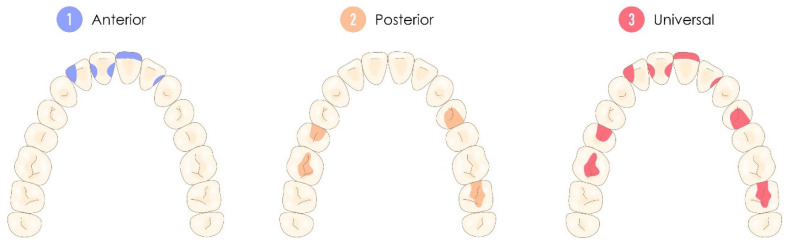
Representation of the location of use classification, considering both the location and the type of restoration class that the resin composite can effectively address. Universal resin composites are therefore considered suitable for use in both the anterior and posterior sectors, depending on their major characteristics.

Finally, other more marginal classifications have been proposed, such as those based on whether a resin composite is used anteriorly, posteriorly or both [47,74]. This classification is indisputably the most clinical of all historical classifications but also the most reductive and the least contributive now that, as we saw earlier, the field of use for flowable resin composites has drastically increased as their mechanical properties have improved. Thus, a resin composite with properties that could be used in both the anterior and posterior sectors was called “universal”, whereas others had their field of use specified by the terms “anterior” or “posterior” [47,74]. Figure 5 illustrates this historical classification according to the type of restoration class these resin composites can achieve.

## 3. Proposed Modular Classification Based on the Clinical Features of Direct Dental Resin Composites

**Figure 6:**
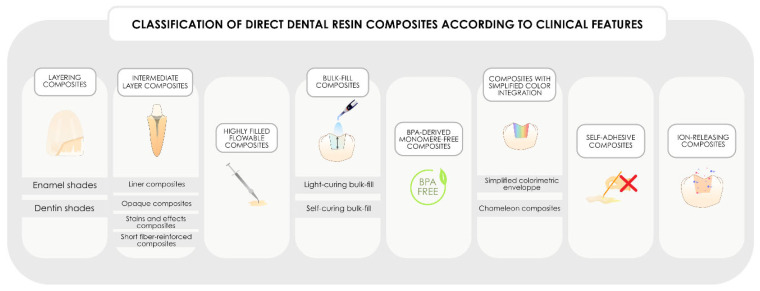
Classification of direct dental resin composites according to their clinical features. Importantly, as the future of chemical development aims to combine multiple clinical features within a single formulation, this classification may include several clinical characteristics to describe the same formulation.

Faced with this need for a more “clinical” classification, currently, a need exists to propose and formalize a classification of resin composites based on their potential clinical characteristics. This classification is all the more important, as new formulations now offer the possibility of combining several clinical characteristics in a single formulation. As a result, a given resin composite may feature one or more of the characteristics listed in this classification to characterize it. The proposed modular classification, which is developed in the remainder of this article and is based on eight major clinical families and a total of 14 subcharacteristics, is summarized in Figure 6. In the future, if new disruptive features were to appear, it would be sufficient to simply add them to the classification, without affecting the use of the others presented, hence the concept of modular classification with multiple characteristics possible for a same formulation.

### 3.1. Layering Resin Composites (Enamel and Dentin Shades Available)

Historically, this family encompassed universal viscous resin composites, regardless of the structure or distribution of their fillers, to return to previous classifications. Recently, however, we have introduced flowable resin composites within this same category, expanding its scope. The wear resistance and polishing ability of these materials are variable and are more formulation-dependent than structure-dependent [16,71].

The key feature of this family is the extensive range of resin composite shades available within a single formulation, which are meticulously designed to replicate the optical properties of natural teeth, including both the dentin core and enamel shell. These resin composites are intended to be combined to achieve optimal aesthetic and functional outcomes.

**Figure 7:**
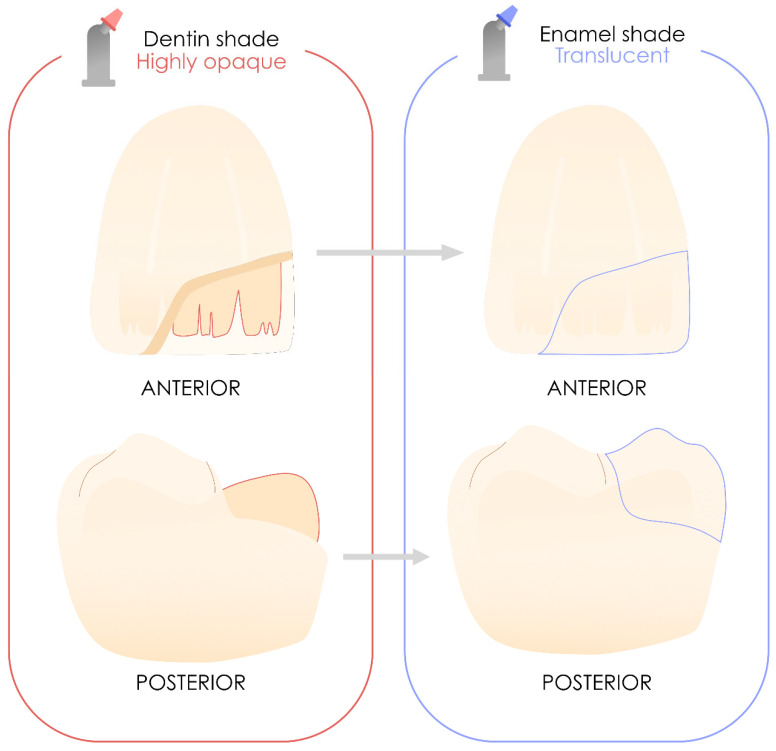
Use of layering resin composites in the anterior and posterior sectors. When properly used, they will deliver the best aesthetic results.

These resin composites include highly opaque, dentin-saturated materials designed for the replacement of dentin structures, as well as translucent enamel materials that closely mimic the natural appearance of teeth. These are still used and recommended for highly aesthetic layering [31,75,76,77]. Thus, they play a significant role in the practitioner’s therapeutic arsenal. Figure 7 illustrates the potential application of these materials in both anterior and posterior restorations using layering techniques, although their use in posterior sectors remains limited among clinicians [78,79].

The most common formulations currently available are Filtek Z350XT (3M ESPE, St. Paul, MN, USA), Tetric EvoCeram (Ivoclar, Schaan, Liechtenstein), Estelite Asteria (Tokuyama, Tokyo, Japan), Clearfil Majesty ES-2 (Kuraray, Tokyo, Japan), IPS Empress Direct (Ivoclar, Schaan, Liechtenstein), and Enamel Plus HRi (Mycerium S.p.A, Avegno, Italy).

### 3.2. Intermediate Layer Resin Composites

This family of resin composites includes formulations that are unsuitable for exposure in the oral environment or load-bearing areas due to inadequate optical or mechanical properties or insufficient resistance to hydrolysis. Four distinct subcategories can be identified within this family.

#### 3.2.1. Liner Resin Composites

Liner resin composites are composed of flowable formulations used for their self-leveling properties, especially in class I, class II and class V cavities. They represent historical flowable resin composites with insufficient mechanical properties for use in areas of mechanical stress and are used to improve the adaptation of future viscous resin composite layers to cavity walls and dissipate the polymerization stresses of subsequent layers through their low elastic modulus (such as the example given for flowable resin composites in Figure 4) [80,81,82]. Although this positive clinical or microleakage effect is debated in the literature [83], these resin composites are still widely used for this purpose.

They are therefore characterized by a low elastic modulus and extremely low viscosity, optimizing their self-leveling and self-spreading properties. More sporadically, they can also be used in co-polymerization with a viscous composite, which also leads to good clinical results [84,85,86].

The most common formulations currently available are Tetric Evoflow (Ivoclar), G-aenial Flow X (GC Corporation, Tokyo, Japan), Venus Flow (Kulzer, Hanau, Germany) and Ceram.X Spectra ST Flow (Dentsply-Sirona, Constanz, Germany).

#### 3.2.2. Opaque Resin Composites

Flowable or viscous resin composites in this category exhibit an exceptionally high masking ability for underlying structures, surpassing the opacity of dentin shades commonly found in layering resin composites [87,88,89]. However, their masking effect becomes fully apparent only when they are applied at sufficient thicknesses [87,88,89,90].

**Figure 8:**
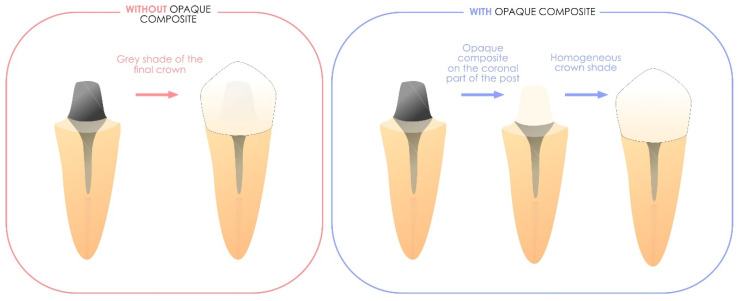
Illustration of the benefits of using a masking liner, including its ability to mask a metallic intended to be permanent. It is also effective for concealing discolored areas of the tooth, such as those following amalgam removal or when discoloration persists in structurally anomalous tissues.

These materials are defined by their high opacity, making them ideal for masking oversaturated dental tissues or metal elements before the application of subsequent direct or indirect restorations. Importantly, they are not designed to be exposed directly to the oral environment. Figure 8 illustrates the clinically relevant applications of these formulations.

The most common formulations currently available are Masking Liner (GC Corporation), Filtek Universal Pink Opaque (3M ESPE) and IPS Empress Direct Opaque (Ivoclar).

#### 3.2.3. Stains and Effects of Resin Composites

**Figure 9:**
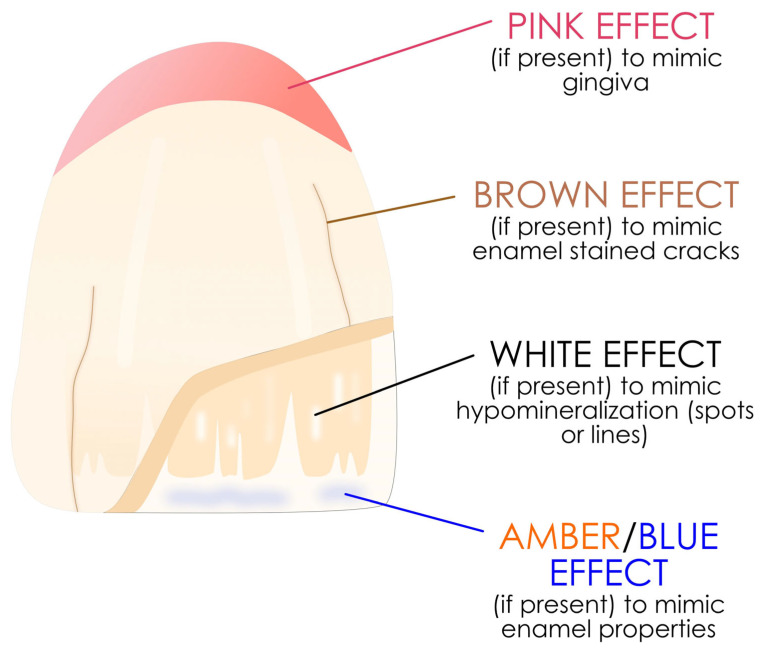
Proper use of these stains on an anterior tooth between the dentin and enamel layers. The concept is simplified but the same in the posterior area.

These resin composites, generally in flowable form (but not exclusively), are incorporated between the enamel and dentin masses of the layering resin composite in very small amounts to restore the effects of translucency, opalescence and groove color. They are not exposed to the oral environment except when they are used to create an illusion of the gingiva. They can also be used on endodontically treated teeth to improved aesthetics [91]. Figure 9 shows where they are able to be used to restore anterior teeth with the layering technique.

The most common formulations currently available are IPS Empress Color (Ivoclar), Amaris (VOCO GmbH, Cuxhaven, Germany), Gradia Gum (GC Corporation) and Venus Color (Kulzer).

#### 3.2.4. Short Fiber-Reinforced Resin Composites

These resin composites are glass-fiber-reinforced resin composites designed to compensate for substance loss in severely damaged teeth and cannot be exposed to the oral environment.

They are available in either flowable or viscous forms, with randomly dispersed glass fibers embedded within the resin matrix. For fibers to function effectively as supports in a polymer matrix, tress transfer from the matrix to the fibers must be optimal. This property requires that the fibers reach a sufficient quantity [92] and a minimum length, known as the “critical fiber length” [93], from which mechanical stress is effectively transferred, thus improving the overall properties of the material. For effective reinforcement, the fiber length must be at least 50 times greater than the fiber diameter [94].

The role of short fiber-reinforced resin composites is to act as a fuse, effectively halting cracks or confining fracture propagation within itself, thereby preserving the tooth from catastrophic failure more efficiently [95,96,97,98]. Owing to their physical and mechanical properties, these resin composites can be used for the biomimetic replacement of dentin in large cavities and endodontically treated teeth to reduce the risk of catastrophic failure of the teeth [99]. However, these short fiber-reinforced resin composites should always be covered with another resin composite to prevent hydrolysis between the fibers and the resin matrix, justifying their classification among intermediate layer resin composites [100].

**Figure 10:**
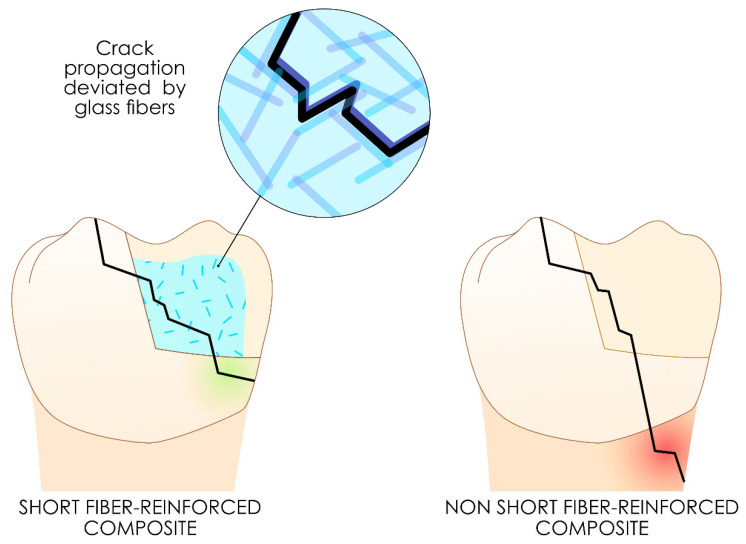
Proposed concept of crack deviation to a more favorable pattern using these short fiber-reinforced resin composites.

Figure 10 illustrates the proposed concept of crack deviation to a more favorable pattern with these resin composites.

The most common formulations currently available are Ever-X Posterior (GC Corporation) and Ever-X Flow (GC Corporation).

### 3.3. Highly-Filled Flowable Resin Composite, Also Called High-Performance Flowable Resin Composite

These flowable resin composites, often mistakenly referred to as “injectables” due to their specific trade name, can be used to compensate for substance loss in anterior or posterior teeth under occlusal stress [101]. Additionally, they are also suitable for use in injection molding techniques [102,103]. These broad indications are supported by experimental evidence showing that these flowable resin composites with an increased filler ratio exhibit, on average, enhanced mechanical properties—specifically, greater flexural strength and elastic modulus. More importantly, they demonstrate significantly improved wear resistance, making them well-suited for posterior applications [71,104]. It is these characteristics that explain their name, which is inconsistently found in publications.

Innovations in the filler size and silanization treatment are believed to underlie these improvements [71]. These resin composites are often perceived by practitioners as flowable resin composites with low spreading power due to their viscosity (i.e., not self-levelling, allowing occlusal sculpting or injection treatments). Indeed, although this characteristic is present in the majority of formulations, some formulations have very high flowability. However, care should be taken when these materials are used with high C-factors, as these resin composites exhibit greater polymerization stress than other formulations [105,106].

**Figure 11:**
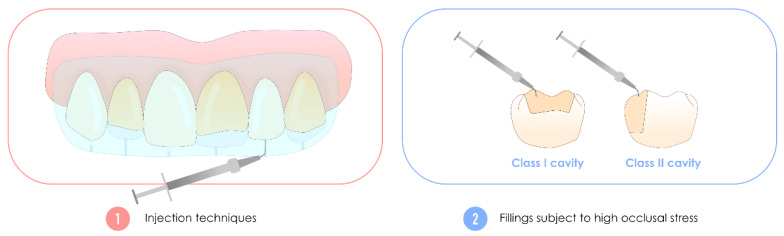
Clinical illustration of the additional clinical indications of highly filled flowable resin composites compared with historical flowable resin composites used as liners. Two major new features are the need to perform extensive fillings, such as posterior Class I and Class II cavities, which are subject to high occlusal stress, and their use for injection treatments.

Figure 11 shows the major clinical indications of these highly filled flowable resin composites compared with the historic flowable resin composites used as liners.

The most common formulations currently available are G-aenial Universal Injectable (GC Corporation), Clearfil Majesty ES Flow (Kuraray) and Filtek Supreme Flowable Restorative (3M ESPE).

### 3.4. Bulk-Fill Resin Composites

Two distinct subcategories can be identified for this type of composite, the characteristics of which allow resin composite increments greater than 2 mm, as is the case for most formulations, to ensure good polymerization and control of polymerization stresses: light-curing resin composites with an increased polymerization depth and self-curing resin composites with a theoretically infinite polymerization depth.

#### 3.4.1. Light-Curing Bulk-Fill Resin Composites with an Increased Polymerization Depth

These resin composites, which exist in viscous or flowable form, appeared more than 10 years ago and now have an important clinical record. Given their “fast dentistry” ability, they are now widely used for rehabilitation in numerous studies reporting clinical performances comparable to those of historical resin composites [107,108].

They are characterized by the fact that they can be used with much greater photopolymerization depths (4 to 6 mm for brands) than other resin composites.

**Figure 12:**
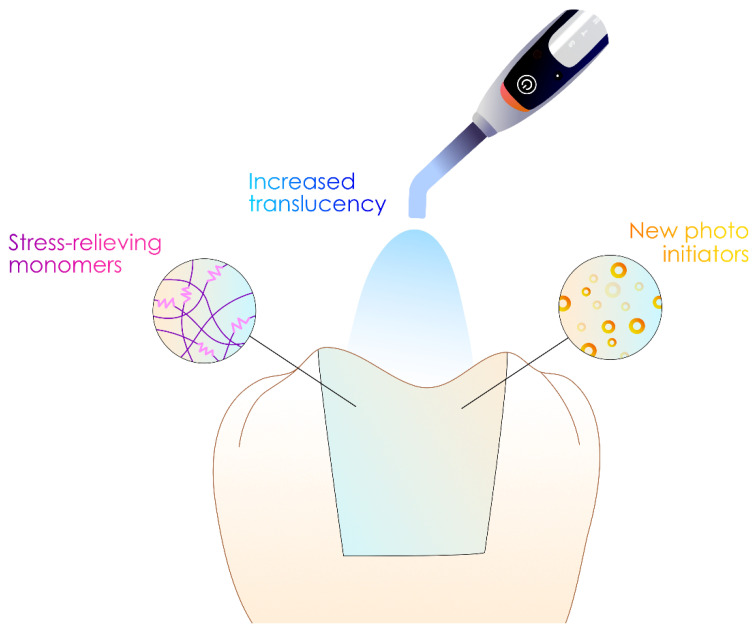
Modifications of the resin composite to increase the photopolymerization depth without developing excessive stresses.

They are the product of chemical innovations that have increased the depth of polymerization without increasing the polymerization stresses generated by their setting. These properties were facilitated by increasing their translucency and incorporating new photoinitiators into their composition (to increase the polymerization depth), as well as incorporating stress-relieving monomers such as modified UDMAs or chain transfer agents into their monomer composition [29,109,110,111], as shown in Figure 12. This latest technology even enables the creation of bulk-fill resin composites with a light-curing time reduced to three seconds [112,113].

The most common formulations currently available are SDR (Dentsply-Sirona), Venus Bulk Flow (Kulzer), Filtek Bulk Fill Flow (3M ESPE), Tetric EvoCeram Bulk-Fill (Ivoclar), Tetric PowerFill (Ivoclar) and Tetric PowerFlow (Ivoclar).

#### 3.4.2. Self-Curing Bulk-Fill Resin Composites with Unlimited Polymerization Depth

In recent years, self-curing formulations have regained popularity in direct restorations. The earliest resin composites were derived from a paste–paste mixture [114] and self-curing (or dual-curing) resin composite; however, they have been available for decades and are still used by some practitioners for fabricating resin composite build-ups and bonding intraroot canal posts [115,116,117]. In recent years, however, the new self-curing materials arriving on the market have tended to be from other families and have been mostly limited to material families such as glass ionomer cements (GICs) and resin-modified glass ionomer cements (RM-GICs), although some have been mistakenly classified as resin composites [32,71]. A resinous formulation must have a water-free composition to be classified as a resin composite [32].

The most common formulations currently available for these historic formulations are Multicore Flow (Ivoclar), ParaCore Automix (Coltene/Whaledent AG, Altstätten, Switzerland), Gradia Core (GC Corporation) and Luxacore Z (DMG, Hamburg, Germany).

These resin composites can polymerize in the absence of photopolymerization, i.e., by a chemopolymerization reaction alone (and possibly an accessory photopolymerization reaction).

However, new self-curing resin composites have recently been added to the range of materials available to perform direct single-increment restorations, alongside a touch-curing primer to bond to the tooth structure. Although potentially promising in the clinic, their results appear to be limited [118,119,120,121].

The most common formulations currently available for these new formulations are as follows: Cention N (Ivoclar), Cention Forte (Ivoclar) and Stela (SDI, Bayswater, Australia). These three materials are also capable of ion release under certain conditions and will be described in greater detail later.

### 3.5. BPA-Derived Monomer-Free Resin Composites

Recently, the increasing use of resin-based dental materials in the oral cavity has raised concerns regarding the biocompatibility and safety of the resin matrix components [122,123,124]. The possible release of bisphenol A (BPA) impurities by resin composites containing their derivatives and their toxicity at low doses are highly debated and growing problems [125,126,127]. Bisphenol A derivatives used in resin composites mainly include BisGMA, bisphenol A dimethacrylate (BisDMA), ethoxylated bisphenol A glycol dimethacrylate (BisEMA), polycarbonate-modified BisGMA (PC BisGMA), and 2,2-bis[(4-methacryloxy polyethoxy)phenyl]propane (BisMPEPP) [128].

These concerns have led to the development of so-called “BPA-free” resin composites in a flowable or viscous form, which generally contain UDMA derivatives as a replacement matrix [128]. Although these resin composites may release other monomers [128,129,130], the absence of possible BPA release from their monomeric matrix characterizes them. The clinical and in vitro performance of these formulations appear to be similar to those containing BPA derivatives [131].

The most common formulations currently available are Venus Pearl (Kulzer), Venus Diamond (Kulzer), G-aenial Anterior (GC Corporation), SDR (Dentsply-Sirona), Omnichroma (Tokuyama), ENA HRi Enamel Bio Function (Mycerium) and Purefill 2 Bio + (Elsodent, Villebon-sur-Yvette, France).

### 3.6. Resin Composites with Simplified Color Integration

These resin composites (although available in both viscous and flowable formulations) represent a natural evolution of the “historic” viscous layering resin composite to limit the number of shades (enamel–dentin) used for the efficient integration of the restoration. Although a consensus definition is currently unavailable, they can be defined as resin composites whose optical properties enable them to adapt more closely to the surrounding tooth color. With a limited number of syringe(s)/compule(s), these resin composites with simplified colorimetric integration can be used to meet all clinical situations from a colorimetric perspective without the need for complex layering.

Two distinct subcategories can be identified for this family featuring the simplified aesthetic integration of resin composites: multisyringe resin composites with simplified color envelopes based on luminosity, akin to the “body” resin composites that were historically offered as laminated resin composites, and single-syringe chameleon resin composites with high translucency that are based on the structural color.

Some syringes, regardless of their category, are sometimes combined with an opaque “blocker” for certain clinical cases because of their increased translucency, such as to mask discolored substrates or, in the case of anterior restorations, to mask the buccal base [75,132].

#### 3.6.1. Multi-Syringe Systems with Simplified Colorimetric Envelope, Also Called “Cloud-Shade” Resin Composite

The simplified color envelope, simplified shade resin composite or group-shade resin composites are multisyringe systems designed to provide group-based color adaptability (based on the Vita Classical shade guide) [133,134,135]. These materials are produced with calibrated luminosity, which is a fixed brightness level that corresponds to the value range of a specific natural tooth shade. This property is particularly important, as the human eye is more sensitive to variations in value than to changes in chroma or hue [136]. Instead of relying on dynamic optical blending such as the chameleon resin composite detailed below, they achieve versatility by maintaining a balanced translucency associated with a pigment color [137]. Manufacturers often categorize these resin composites into a few “envelopes”, such as light, medium, and dark. The opacity of these materials is intermediate between the enamel and dentin masses of layering resin composites [137].

They are characterized by a more restricted (but still adaptable) range of shades, mostly based on calibrated luminosity, in contrast to layering resin composites. These materials rely on pigmented colors, enabling the creation of single resin composite dental restorations with a simplified shade-matching process.

Clinically, these materials are efficient at restoring an acceptable degree of compromise between the anterior and posterior teeth when time efficiency and predictability are needed [134].

The most common formulations currently available are G-aenial A’chord (GC Corporation), Tetric Prime (Ivoclar), TPH Spectra LT (Dentsply-Sirona), Filtek Universal Restorative (3M ESPE), Filtek Suprem EasyMatch Universal Restorative (3M ESPE) and SimplyShade Universal (Kerr, Orange, CA, USA).

#### 3.6.2. Monosyringe Systems, Also Called Chameleon Resin Composites

Chameleon resin composites are advanced resin composites designed to achieve seamless aesthetic integration by blending dynamically with the surrounding tooth structure [75,138]. They rely heavily on high translucency to transmit and reflect light from adjacent dental tissues, creating a “fusion effect” [139]. This property is complemented by a phenomenon known as structural color (without pigments) [75,140], which is based on scattering due to a controlled filler size (approximately 260 nm) and a homogeneous distribution in the matrix [141,142]. The combination of high translucency (adaptation to real tooth color) and structural color (statistical shade) makes them more effective when the cavity to be restored has many tooth walls [132,141]. A key feature of chameleon resin composites is their broad optical adaptability, allowing them to match a wide range of shades with a unique syringe. However, their high translucency can be a limitation in cases where the underlying substrate is dark or opaque, as these materials may not sufficiently mask the background [132,143].

They are characterized by a single-shade formulation, high translucency, and a reliance on structural color, enabling the creation of single resin composite dental restorations that naturally blend with the surrounding tooth structure.

Their performance is highly dependent on the environmental lighting and the natural color of the neighboring tooth structures, but when they are used in the right indications, they deliver excellent clinical performance [144,145].

The most common formulations currently available are Omnichroma (Tokuyama), Vittra Unique APM (FGM Dental Group, Joinville, Brazil), Venus Pearl One (Kulzer), Zen Chroma One Shade (President Dental GmbH, Munich, Germany), Ecosite One (DMG), Beautifil UniShade (Shofu Inc., Kyoto, Japan), ONE Shade (SDC, Shanghai, China), Clearfil Majesty ES-2 U (Kuraray), Venus Diamond One (Kulzer), Clearfil Majesty ES-2 Universal (Kuraray) and Essentia Universal (GC Corporation).

**Figure 13:**
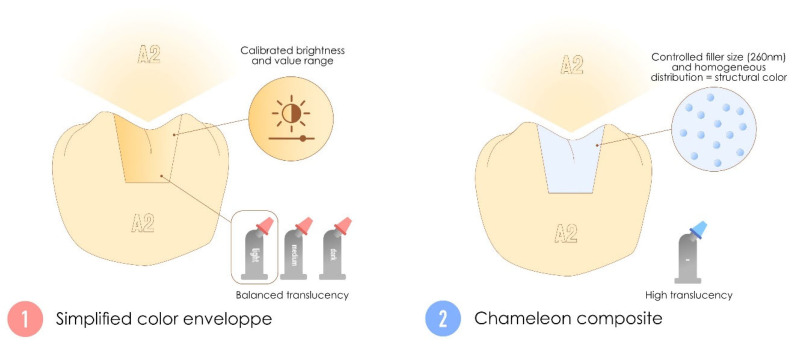
Differences between simplified color envelope resin composites and chameleon resin composites. Although these two materials arrived on the market in the same time frame, they have very different indications and operating systems.

Figure 13 illustrates the conceptual differences between a resin composite with a simplified colorimetric envelope and a chameleon resin composite.

### 3.7. Self-Adhesive Resin Composites

Due to their flowable consistency (required for sufficient wettability and adhesion to hard dental tissues), these resin composites have been proposed as alternatives to the adhesive/composite pairing used in direct restoration [146], consistent with the concept of continuing to simplify procedures for clinicians. Their self-adhesive effect is achieved through the action of monomeric acid groups similar to those found in universal adhesives, such as 4-methacryloxyethyl trimellitic acid (4-META), glycerophosphate dimethacrylate (GPDM) and 10-methacryloyloxydecyl dihydrogen phosphate (MDP) [147,148].

They are characterized by their self-adhesion properties to hard dental tissue without the need for an adhesive system or a tooth primer.

Their actual in vitro performance has been shown to be inferior to that of the adhesive–composite pair in terms of adhesive [147,149] or mechanical properties [150]. However, their short-term clinical performance appears good in the small number of studies available [148,151,152].

The most common formulations currently available are Fusio Liquid Dentin (Pentron, Wallingford, CT, USA), Constic Flow (DMG) and Vertise Flow (Kerr).

### 3.8. Ion-Releasing Resin Composites

The idea of creating an ion-releasing resin composite capable of inducing the remineralization of dental tissue or limiting the risk of inducing dental caries is not new, as it was already the concept behind compomers or giomers. The concept of their effect is based on a phenomenon of water absorption leading to the degradation of inorganic charges similar to those contained in GICs and RM-GICs and thus to the release of ions, notably fluorine [32]. Studies indicate that their ion release is insufficient for any significant clinical benefit, whereas the water absorption inherent to their chemistry progressively compromises their mechanical properties over time [5,32,153,154]. Moreover, using these formulations in conjunction with an adhesive system on the tooth would logically limit their potential actions [155].

The most common formulations available for compomers and giomers are Dyract Extra (Dentsply-Sirona), Compoglass (Ivoclar), F2000 (3M ESPE), Ionolux (Voco), Beautifil II (Shofu), and Beautifil flow Plus (Shofu).

This family of resin composites has regained popularity with the introduction of new formulations branded as “alkasite”, “smart composites” or even “bioactive composites” based on a similar concept [32,71]. Compared with other resin composite families, they are still too recent to determine their performance, but they perform well in the short term [80,153,156,157].

**Figure 14:**
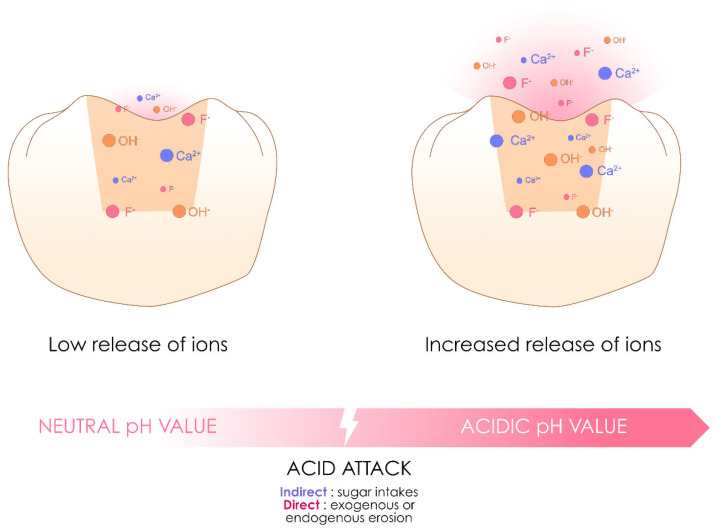
Inspired by the diagram proposed by Ivoclar for the mechanism of action of Cention Forte, this image illustrates the concept of the currently available ion-releasing resin composites, which are capable of ion release, the characteristics of which vary according to the formulation and can be accentuated under specific intraoral conditions, notably acidity.

Resin composites in this family are therefore characterized by potential ion release via water sorption and depend on the conditions of the oral environment (such as the presence of a decrease in pH). Figure 14 illustrates the operating principle of these ion-releasing resin composites.

The most common formulations currently available are Cention N (Ivoclar), Cention Forte (Ivoclar) and Stela (SDI). Notably, these formulations have already been used for self-curing resin composites, confirming that the same formulation can belong to several families.

## 4. Conclusions

This review presents a novel clinical classification for resin composites, addressing the shortcomings of traditional systems that focus solely on the inorganic structure, viscosity, or monomer composition. By prioritizing the clinical characteristics, the proposed modular approach provides greater clarity for researchers and practitioners navigating the increasingly diverse range of composite formulations. This system highlights key features, such as bulk-fill capabilities, layering versatility, and ion-releasing properties, streamlining both clinical decision-making and formulation selection. Its relevance is particularly significant, as modern products often combine multiple characteristics, blurring traditional categorical boundaries. While the classification enhances our understanding and the application of resin composites, long-term clinical validation of these products remains essential. Furthermore, continuous monitoring of future innovations will be critical, as emerging developments may refine and reshape the applicability of this classification system.

## Data Availability

No new data were created or analyzed in this study.
